# Chemical and Biological Significance of Oenothein B and Related Ellagitannin Oligomers with Macrocyclic Structure

**DOI:** 10.3390/molecules23030552

**Published:** 2018-03-02

**Authors:** Takashi Yoshida, Morio Yoshimura, Yoshiaki Amakura

**Affiliations:** 1 College of Pharmaceutical Sciences, Matsuyama University, 4-2 Bunkyo-cho, Matsuyama, Ehime 790-8578, Japan; xp769b@bma.biglobe.ne.jp (T.Y.); myoshimu@g.matsuyama-u.ac.jp (M.Y.); 2 Okayama University, Okayama 701-1152, Japan

**Keywords:** oenothein B, ellagitannin, macrocyclic oligomer, Onagraceae, Myrtaceae, Lythraceae, antioxidants, antitumor effect, immunomodulatory effect, anti-inflammation

## Abstract

In 1990, Okuda et al. reported the first isolation and characterization of oenothein B, a unique ellagitannin dimer with a macrocyclic structure, from the *Oenothera erythrosepala* leaves. Since then, a variety of macrocyclic analogs, including trimeric–heptameric oligomers have been isolated from various medicinal plants belonging to Onagraceae, Lythraceae, and Myrtaceae. Among notable in vitro and in vivo biological activities reported for oenothein B are antioxidant, anti-inflammatory, enzyme inhibitory, antitumor, antimicrobial, and immunomodulatory activities. Oenothein B and related oligomers, and/or plant extracts containing them have thus attracted increasing interest as promising targets for the development of chemopreventive agents of life-related diseases associated with oxygen stress in human health. In order to better understand the significance of this type of ellagitannin in medicinal plants, this review summarizes (1) the structural characteristics of oenothein B and related dimers; (2) the oxidative metabolites of oenothein B up to heptameric oligomers; (3) the distribution of oenotheins and other macrocyclic analogs in the plant kingdom; and (4) the pharmacological activities hitherto documented for oenothein B, including those recently found by our laboratory.

## 1. Introduction

**Figure 1:**
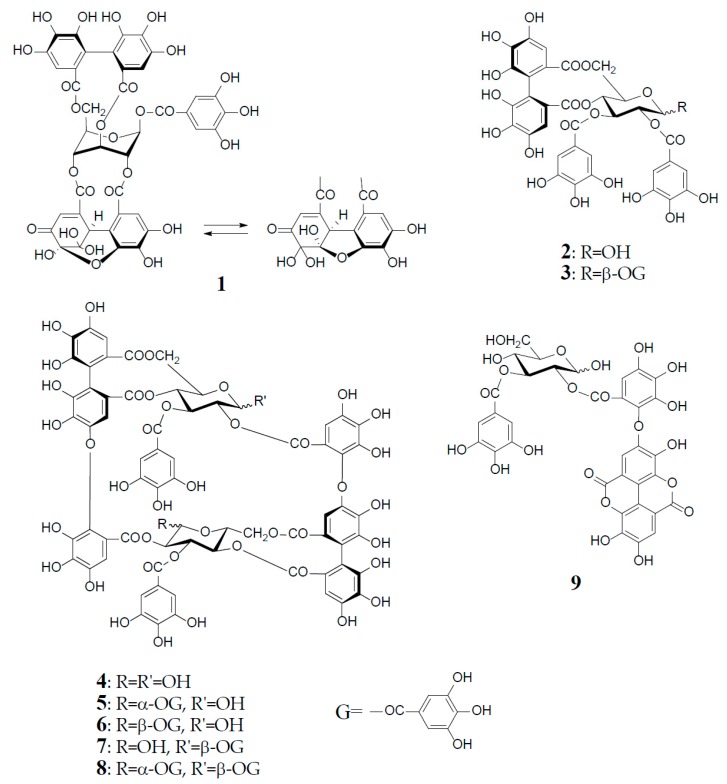
Structures of geraniin (**1**), tellimagrandin I (**2**), and II (**3**), oenothein B (**4**), woodfordin C (**5**), eugeniflorin D_1_ (**6**), cuphiin D_2_ (**7**), cuphiin D_1_ (**8**), and oenothein C (**9**).

Antioxidant polyphenols in medicinal plants, foods, and fruits are currently acknowledged as important beneficial constituents that reduce the risk of life-related diseases closely associated with active oxygen damage, such as cancers, arteriosclerosis, diabetes, and coronary heart diseases, and have been explored as plausible chemopreventive agents for the human healthcare market. Polyphenols have thus received increasing attention for the discovery and development of their new physiological functions. Among various types of antioxidant plant polyphenols are low molecular weight compounds, represented by flavonoids and lignans, and higher molecular weight polyphenols classified as tannins. Vegetable tannins are classified into two large groups: (1) condensed tannins (proanthocyanidin polymers and oligomers); and (2) hydrolysable tannins, which are subgrouped into gallotannins (polygalloyl esters of glucose) and ellagitannins, which are characterized as hexahydroxydiphenoyl (HHDP) esters of sugar, mostly glucose, as represented by geraniin (**1**), tellimagrandin I (**2**), and II (**3**). In contrast to condensed tannins and gallotannins (Turkish or Chinese gall), which were long recognized in the leather industry [1], ellagitannins in medicinal plants had been little studied before the discovery of geraniin (**1**) from a Japanese folk medicine, *Geranium thunbergii* (Geraniaceae), by Okuda’s group in 1976 [2,3]. Since 1976, remarkable progress in the field of ellagitannin chemistry, promoted by the development of high resolution NMR and MS spectrometers and new separation methods, has led to the isolation and characterization of more than 500 ellagitannins with diverse arrays of structures from the traditional medicines long used in Japan, China, and South East Asia. The structural diversity of the ellagitannins are brought by various oxidative modifications of the HHDP group producing dehydroellagitannins, such as **1** or by intermolecular C‒O oxidative coupling(s) among multiple molecules, leading to oligomeric ellagitannins [4,5,6,7]. The first dimeric ellagitannin encountered in nature was agrimoniin from *Agrimonia pilosa* (Rosaceae), which was characterized as a dimer of potentillin (1-*O*-galloyl-2,3/4,6-di-*O*-(*S*)-HHDP-α-d-glucose), produced through the formation of a dehydrodigalloyl linking unit by intermolecular C‒O oxidative coupling between two galloyl groups at C-1 [8]. Among the more than 300 oligomers, up to heptamer, reported after the discovery of agrimoniin, oenothein B (**4**) is a unique macrocyclic ellagitannin dimer, which is biogenetically produced by double C‒O couplings of two molecules of tellimagrandin I (**2**), as illustrated in Figure 1. 

Oenothein B (**4**) was first isolated as a major component from the leaves of *Oenothera erythrosepala* (Onagraceae) in 1990 [9], and later found widely distributed in other plant species belonging to Myrtaceae and Lythraceae, as well as Onagraceae [5,6,10,11]. It was an important leading compound that made easier the structure elucidation of analogous oligomers co-occurring in various plant species. Furthermore, oenothein B and related oligomers have been reported to exhibit a variety of in vitro or in vivo physiological activities beneficial to human health.

This review summarizes the structural characteristics of oenothein B (**4**) and related oxidized metabolites, up to heptameric oligomer, found in medicinal plants and their diverse biological functions hitherto reported, including those discovered recently in our laboratory. This review provides a better understanding of the significance of those antioxidant tannin constituents in medicinal plants, which may lead to future developments of preventive or therapeutic agents for various chronic diseases associated with oxygen stress by active oxygen species and free radicals.

## 2. Structural Characteristics of Oenothein B 

Oenothein B (**4**), FABMS *m*/*z* 1569 [M + H]^+^, was obtained as an amorphous powder forming an inseparable mixture of theoretically four anomers at two C-1 unacylated glucosyl cores, which caused extreme difficulty in its structure elucidation by spectroscopic analysis. In fact, the ^1^H-NMR spectrum in acetone-*d*_6_-D_2_O recorded at ambient temperature is poorly informative due to severe broadening and multiplication of each proton signal. This spectral feature is characteristic of this type of macrocyclic oligomers owing to the anomerization at each glucose core, and also to a poor flexibility of the macro ring arising from a restricted rotation around the ether linkages of two valoneoyl groups. The structure determination of **4** was performed by spectral and chemical methods, briefly described below.

The ^1^H-NMR measurement at an elevated temperature (40‒50 °C) provided a more informative spectrum, indicating the presence of a predominant anomer with anomeric proton signals at δ 6.20 (d, *J* = 3.5 Hz) and δ 4.48 (d, *J* = 7.5 Hz), due to the α- and β-anomers of glucose-I and II, respectively; however, some of the aromatic and sugar proton signals still broadened, probably due to the poor flexibility of the macro ring. A conclusive clue for the structure elucidation of **4** was brought by the NaBH_4_ reduction at the anomeric centers, which gave a sole tetrahydro derivative with two glucitol cores showing a well-resolved simple NMR spectrum. The spectrum clearly indicated the presence of two each of valoneoyl, galloyl, and glucitol groups as components, as revealed by the characteristic six 1H-singlets and two 2H-singlets in the aromatic region. These units were chemically substantiated by acid hydrolysis of **4**, which produced glucose, and by permethylation followed by methanolysis, which afforded methyl tri-*O*-methylgallate and trimethyl (*S*)-octa-*O*-methylvaloneate in a 1:1 molar ratio. The binding modes of the valoneoyl and galloyl groups on the glucose cores in **4** were determined from the long-range ^1^H‒^13^C shift correlation spectrum of the tetrahydro derivative and identification of partial hydrolysates, including oenothein C (**9**), obtained upon treatment of **4** with hot water. The ^13^C-NMR and CD (large positive Cotton effect at 218‒236 nm) spectra of oenothein B were all consistent with the gross structure (**4**) [9] (Figure 1). 

It is noteworthy that the purity of oenothein B (**4**) is hard to assess by reversed-phase HPLC, because of the appearance of multiple peaks on the chromatograph, depending on the different ratio of the anomers. The LC-MS/MS data for oenothein B reported by Toth et al. might be valuable for its identification [11]. Although expensive, oenothein B is now commercially available as analytical standard, and thus can be used as reference compound for the identification of oenothein B isolated from natural sources, by comparisons of the normal and reversed-phases HPLC with those of the commercial reagent.

**Figure 2:**
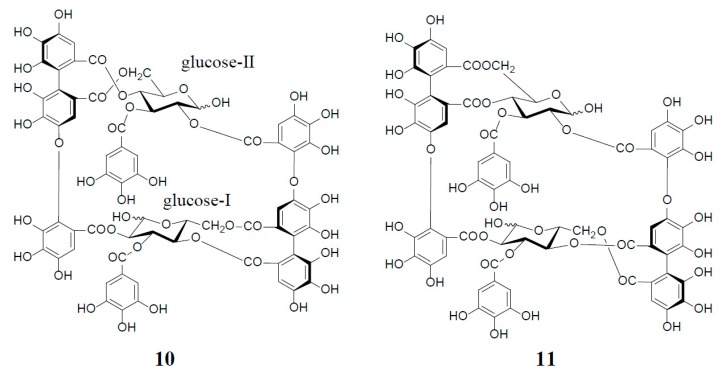
Structures of oenotheins D (**10**) and F (**11**).

Among interesting analogs of oenothein B (**4**) are oenotheins D (**10**) and F (**11**), which were isolated together with **4** (major principle) from the leaves of *Oenothera laciniata*, and characterized as regioisomers of **4**, differing at the binding site of the valoneoyl group linking each monomeric unit, as illustrated in Figure 2 [12]. Contrary to oenothein B (**4**), oenothein D (**10**) displayed a well-resolved ^1^H-NMR spectrum at ambient temperature, and indicated the presence of predominant anomers at each glucose core, as revealed by the unacylated anomeric proton signals at δ 5.89 (d, *J* = 4 Hz; glucose-I) and 4.85 (d, *J* = 8 Hz; glucose-II). The positions of the two valoneoyl moieties in **10** were determined in a similar way to **4**, i.e., long-range ^1^H‒^13^C correlation spectrum and partial degradation in hot water. 

The ^1^H-NMR spectrum of oenothein F (**11**) in acetone-*d*_6_-D_2_O (2 drops) indicated that it exists as a mixture of four anomers at the glucose cores, as shown by the valoneoyl 1H- and galloyl 2H-singlets, each forming four lines in a ratio of ca. 1:2:2:6. It is noteworthy that the relative peak intensity of the four lines for each proton signal changed to ca. 1:4:4:23 after leaving the NMR sample in solution for two days. The ^1^H-NMR spectrum of the most dominant anomer looked like that of a monomeric tannin, namely the appearance of three singlets (δ 6.21, 6.40, and 7.30, each 2H) and one singlet (δ 7.04, 4H) assignable to two valoneoyl and two galloyl units. The sugar proton signals were also apparently those of a monomeric tannin closely similar to those of an α-anomer of tellimagrandin I (**2**). Such a monomer-like ^1^H-NMR spectrum suggested that **11** has a symmetrical structure with a considerably flexible macro ring (Figure 2).

Oenothein B (**4**) and related dimers were also found in plant species of Lythraceae and Myrtaceae, as well as Oenotheraceae. Notably, the lythraceous and myrtaceous plants, unlike Oenotheraceae, produce the galloylated oenothein B together with 4. Woodfordin C (**5**) and eugeniflorin D_1_ (**6**), which are monogalloyl isomers at glucose-I of 4, were obtained from *Woodfordia fruticosa* (Lythraceae), a popular traditional Jamu medicine in Indonesia and Malaysia [13,14], and *Eugenia uniflora* (Myrtaceae), an evergreen fruit tree called Brazilian cherry [15], respectively. The ^1^H-NMR spectrum of 5 (α-gallate at glucose-I), recorded at ambient temperature, displayed broad signals for some of the aromatic and glucose protons, while the spectrum recorded at an elevated temperature (38 °C), which largely contributed to its structure elucidation, was much simpler, and displayed a preferred β-anomer at glucose-II [anomeric proton, δ 4.38 (br. d, *J* = 8 Hz)] [13]. Cuphiin D_2_ (**7**), a β-gallate at glucose-II of 4, was isolated along with a digallate, cuphiin D_1_ (**8**), as well as 4 and 5 from the aerial parts of *Cuphea hyssopifolia* (Lythraceae), which has been used as a folk medicine for treating stomach disorders and oral contraceptive in South and Central Americas [16]. The existence of a dominant α-anomer at glucose-I in 7 (δ 6.18, d, *J* = 3 Hz) was evidenced by the absence of duplicates of any proton signal in the NMR spectra recorded at 40 °C, and also the observation of a single peak in the reversed-phase HPLC. The structural relationship of cuphiins D_1_ (**8**) and D_2_ (**7**) was verified by enzymatic degalloylation of 8, with tannase affording 4, 5, and 7, besides gallic acid (Figure 1).

## 3. Oxidized Metabolites (Dimers and Oligomers) of Oenothein B

**Figure 3:**
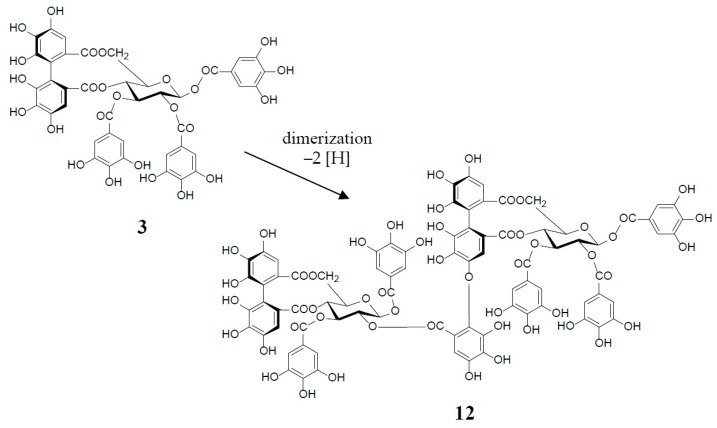
in vitro biosynthesis of cornusiin E (**12**) from tellimagrandin II (**3**) (2 moles).

An old hypothetical biogenesis of ellagitannins [1,4,17] has now been proven by the intensive enzymatic studies of Gross et al. Using crude enzyme preparations from the *Tellima grandiflora* leaves, they demonstrated the in vitro biosynthesis of ellagitannins, which includes an intramolecular C‒C oxidative coupling of pentagalloylglucose to tellimagrandin II (**3**) [18], followed by an oxidative intermolecular C‒O coupling between two moles of 3 to yield a dimeric ellagitannin, cornusiin E (**12**) [19] (Figure 3). These in vitro C‒C and C‒O couplings in the biosynthesis of hydrolysable tannins are thought to occur in vivo through free radical coupling processes involving laccase-type phenolase, with a lower redox potential than those concerned in lignification processes.

**Figure 4:**
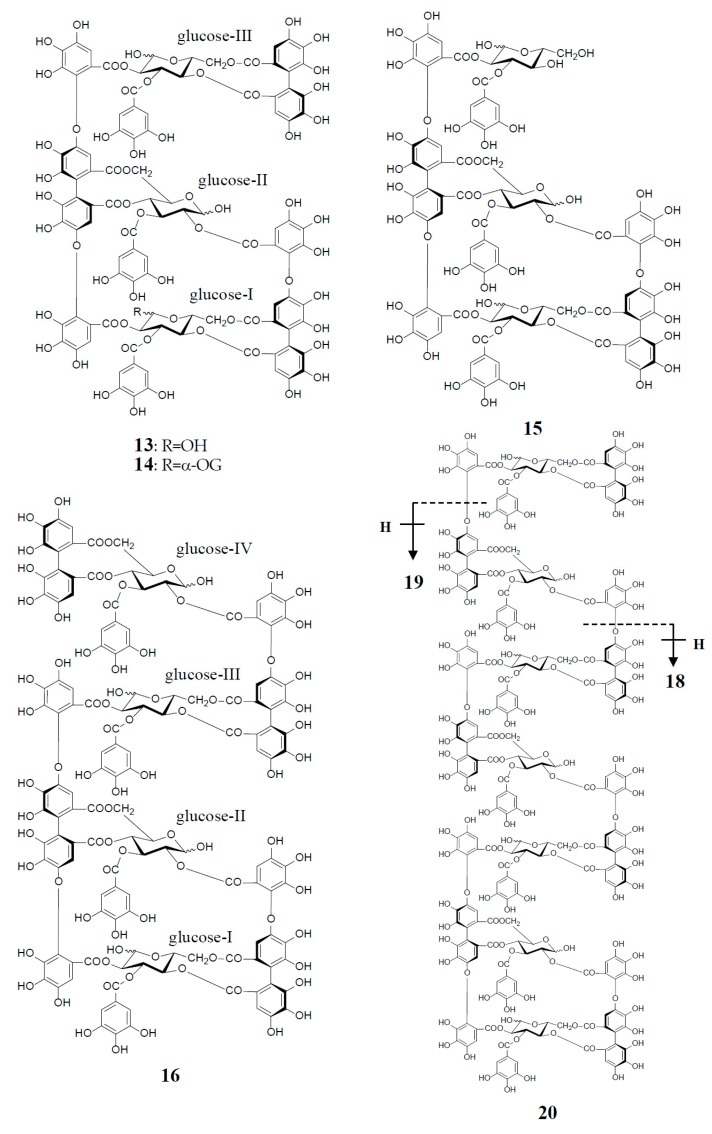

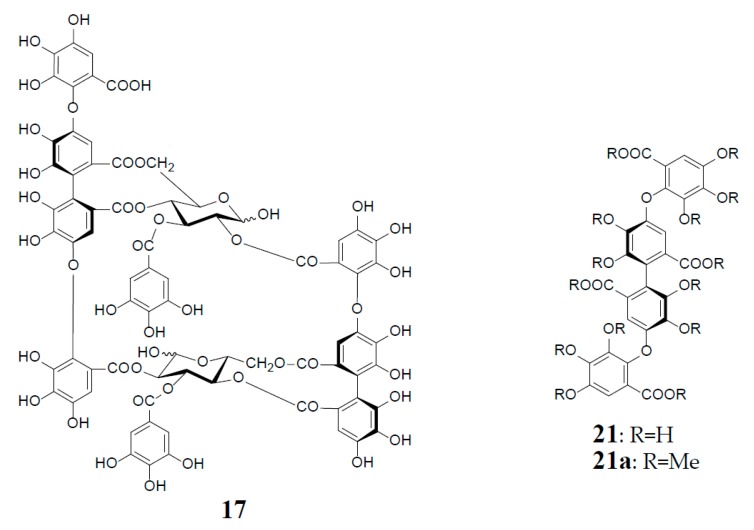
Structures of oenothein A (**13**), woodfordins D (**14**), E (**15**), and F (**16**), pentamer (**18**), hexamer (**19**), and heptamer (**20**), Structures of woodfordin I (**17**) and woodfordinic acid (**21**).

Similar intermolecular oxidative coupling(s) of oenothein B and related dimers with additional monomeric ellagitannin(s) are believed to lead to trimeric and higher oligomeric analogs. Such examples in nature are oenothein A (**13**) from *Oenothera* and *Epilobium* species, and its gallate, woodfordins D (**14**) (trimer) [20], E (**15**) (trimer) and F (**16**) (tetramer), together with woodfordin I (**17**) (dimer) from the *W. fruticosa* flowers [21]. The presence of oenothein B-related oligomers larger than 16 in *Epilobium angustifolium* (willowherb) was recently reported by Salminen et al. [22]. They isolated the oenothein B-based oligomers using preparative HPLC, and characterized them as oenothein B (**4**), oenothtein A (**13**), woodfordin F (**16**), and related pentameric (**18**) to heptameric (**20**) oligomers, chiefly based on the analysis of the fragmentation pattern in the ESI-microTOF-Q mass spectra (negative mode) (Figure 4). The structures of these oligomers were postulated as those produced by the formation of the valoneoyl group through sequential intermolecular oxidative coupling(s) of a galloyl unit at C2 of monomeric tellimagrandin I (**2**) with an HHDP unit of the terminal glucose-IV of woodfordin F (**16**). In the mass spectra, basic fragmentation occurred reversely through the sequential removal of a molecule of tellimagrandin I (**2**) by the oxidative cleavage of an ether bond of the valoneoyl unit from the terminal glucose core, leading to a fragment ion due to the remaining HHDP (*o*-quinone) ester part(s). Quantitative analyses of individual oligomers in the extracts of flowers, leaves, and stems of *E. angustifolium* were successfully performed by ultra-high performance liquid chromatography coupled with tandem mass spectra (UHLC-MS/MS) [22,23]. This analytical method was reported to offer the advantages of good repeatability and sensitivity for an accurate quantification of this class of oligomers, with limits of detection ranging from 0.1 to 1.3 μg/mL.

Woodfordinic acid (**21**), which is the parent acid participating in the linkage of three glucose cores (I–III) in oenothein A (**13**) and woodfordin D (**14**), was characterized as a gallic acid tetramer by spectral analyses (NMR, MS, and CD) of its methylated derivative (21a; C_42_H_46_O_20_) obtained upon permethylation of 14 followed by methanolysis [21]. Its symmetrical structure was evidenced by 2 aromatic proton singlets and 7 methoxy proton signals, and 21 carbon signals comprising of 12 *sp*^2^, 2 ester carbonyl and 7 *sp*^3^ carbon signals in the ^1^H- and ^13^C-NMR spectra, respectively (Figure 4). Woodfordin I (**17**), a dimer possessing the woodfordinoyl group, is likely a catabolic metabolite of 13 and 14. Interestingly, woodfordin I was also isolated from a traditional Chinese medicine, *Chamaenerion* (= *Epilobium*) *angustifolium* [24].

**Figure 5:**
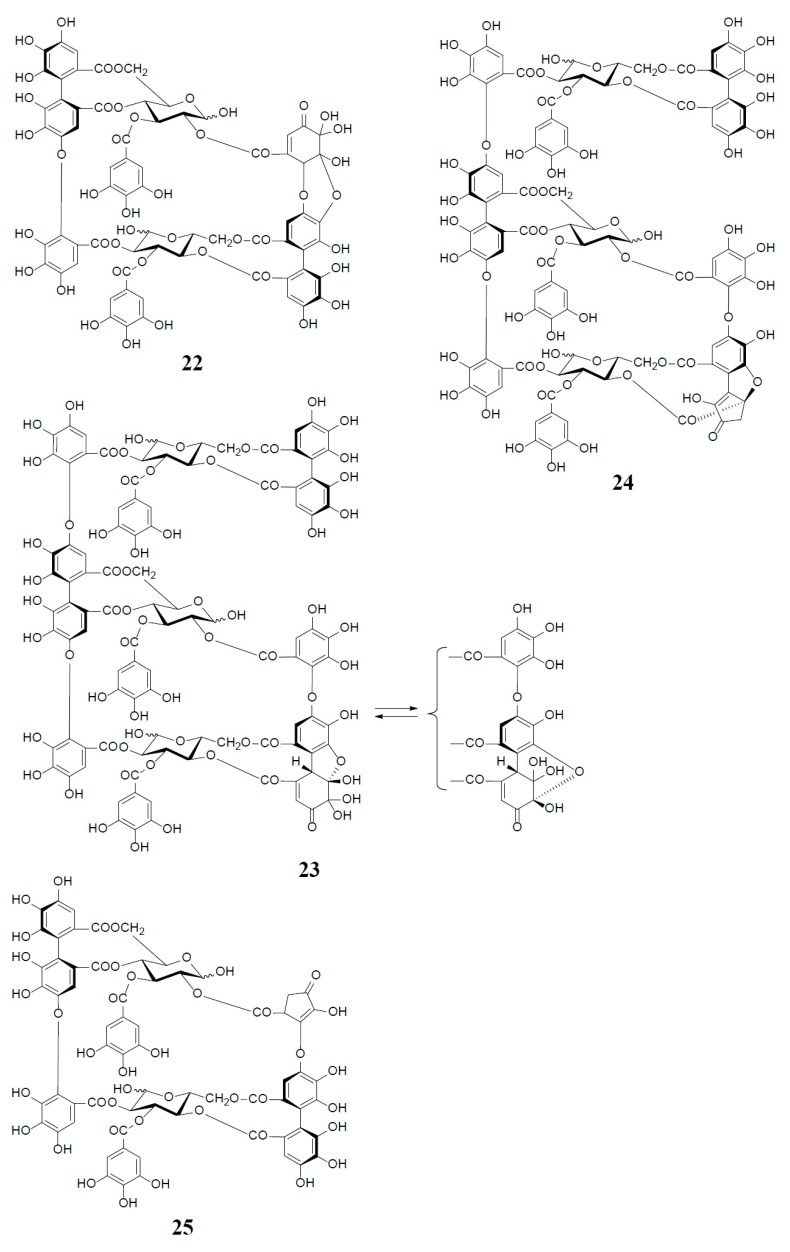
Structures of eugeniflorin D_2_ (**22**), oenotherins T_1_ (**23**), T_2_ (**24**), and eurobustin C (**25**).

Analogs eugeniflorin D_2_ (**22**), and oenotherin T_1_ (**23**) and T_2_ (**24**), all containing an oxidized valoneoyl group, were found in *Eugenia uniflora* [15] and *O. tetraptera* [25,26], respectively. The structural confirmation of oenotherin T_1_ (**23**) was conducted by the Na_2_S_2_O_4_ reduction of the isodehydrovaloneoyl group affording oenothein A (**13**), similar to the conversion of a dehydrohexahydroxyl group to an HHDP group [3]. Notably, in contrast to many *Oenothera* species producing mainly oenothein A (**13**) and B (**4**), the most abundant constituent of *O. tetraptera* was oenotherin T_1_. On the other hand, eugeniflorin D_2_ (**22**), with a dehydrovaloneoyl group isomeric to that in oenotherin T_1_ (**23**), was also found in the leaves of *Eucalyptus cypellocarpa* [27] and *Myrtus communis* of Myrtaceae [28]. Eurobustin C (**25**), isolated from *Eucalyptus robusta* [6], as well as oenotherin T_2_ (**24**), had a new unique linking unit in place of the valoneoyl group, as shown in Figure 5.

In a study on the production of ellagitannins by callus cultures, Taniguchi et al. reported the establishment of callus tissues induced from the *Oenothera laciniata* leaves, which yielded large amounts of oenotheins A (**13**) and B (**4**), as well as oenotherin T_1_ (**23**) [25,29]. It is noteworthy that oenothein B content (65 mg/g dry wt) in the calli cultured on modified Linsmaier–Skoog’s medium was 1.8 times higher than that of intact leaves [29].

## 4. Distribution of Oenothein B and Its Analogs 

**Figure 6:**
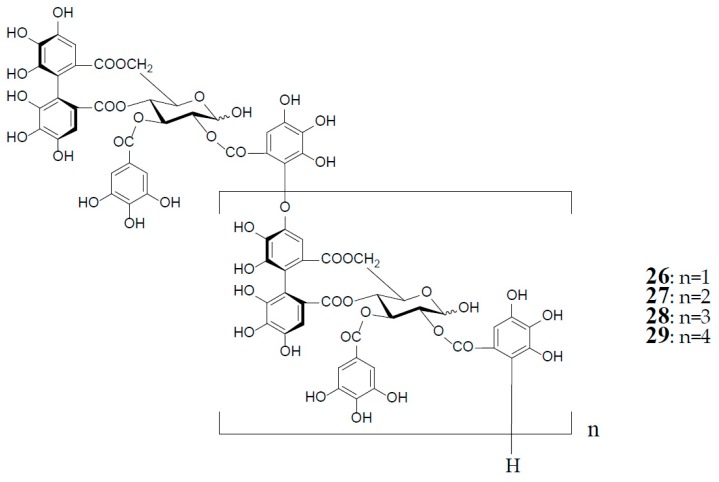
Structures of eucalbanin B (**26**), eucarpanin T_1_ (**27**), pomegraniin A (tetramer) (**28**), and B (pentamer) (**29**).

As described earlier, oenotheins A (**13**) and B (**4**) have been isolated as main ingredients accompanying various analogs from the plant species of Onagraceae, Lythraceae, and Myrtaceae [5,10,11,20,30,31]. The distribution of oenothein B (**4**) in further species of these plant families was examined by HPLC to reveal its considerable wide occurrence, particularly in *Eucalyptus* species of Myrtaceae [30,32]. The oenothein B-containing plants reported so far are summarized in Table 1. Recently, the dried pericarps of *Punica granatum* belonging to Lythraceae (Punicaceae) were reported to produce oenothein B, along with new tellimagrandin I-based linear oligomers, pomegraniin A (tetramer) (**28**) and B (pentamer) (**29**), as well as eucalbanin B (dimer) (**26**) and eucarpanin T_1_ (trimer) (**27**), which were first isolated from the leaves of *Eucalyptus alba* [33] and *E. cypellocarpa* [27] (Myrtaceae), respectively [34] (Figure 6). Although in the classical plant taxonomy Punicaceae belonged to its own family, it is currently included in the Lythraceae family in the phylogenetic system APG III [35]. It is chemotaxonomically interesting that the oligomeric ellagitannins of *P. granatum* showed close resemblance with those of the genera *Cuphea*, *Lythrus*, and *Woodfordia*, which are closely related genera in this family [36], although *P. granatum* is distinguished from the species of the other genera in the elongation mode of the monomers; that is, the presence of an oenothein B-based trimer (**13**) in the latter three, or absence in the former.

## 5. Biological Activities of Oenothein B and Related Oligomers 

Numerous medicinal plants rich in tannins have long been used worldwide as folk medicines or traditional medicines for various purposes, represented by antidiarrheic, hemostatic, and the treatment of gastrointestinal disorders, wound healing, and skin stress [4,7]. The active components of these plant extracts responsible for such therapeutic effects were ascribed to the tannins (large molecular polyphenols), which were long recognized to have non-specific binding ability with proteins (astringency), inducing a peristaltic action. However, the remarkable progress in the structural characterization of ellagitannin constituents in those medicinal plants since the 1980s has enabled studies on various pharmacological activities of individual tannin constituents with defined structures. As a result, diverse biological effects, such as antioxidant, antimicrobial, antitumor, antiulcer, and anti-inflammatory effects, have been found by various in vitro and in vivo studies [4,6,7] and citations therein [41]. The efficacies of such biological activities have been reported to be largely dependent on the difference of types or structures of tannins and related polyphenols, and on their concentrations. Advance in the structural study of ellagitannins also has enabled investigation of the interaction between structure-defined ellagitannins and certain proteins, amino acids, or metals. These studies revealed that tannin–protein complex formations are not due to nonspecific binding with proteins, as previously thought, but largely dependent on the structure and concentration of tannins and targeted proteins. Recently, the importance of molecular size and structural flexibility of ellagitannins in the interaction with bovine serum albumin was emphasized, based on the thermodynamic study of the interaction using isothermal titration calorimetry and fluorescence spectroscopy [42]. 

As oenothein B (**4**) constitutes a unique class of ellagitannins in its macrocyclic structure with limited flexibility of rotational bond, and also in its high content in many medicinal *Oenothera*, *Epilobium*, and *Eucalyptus* species, its biological activities have been widely studied [5,6,7,39,43]. Among such pharmacological effects, hitherto documented for oenothein B and its analogs, this review summarizes selected papers, reporting (1) antioxidant and anti-inflammation activity; (2) antitumor activity; (3) immunomodulatory effects; and (4) antimicrobial effects, including our recent findings.

### 5.1. Antioxidant and Anti-Inflammation Activity

The active oxygen damage or formation of reactive oxygen species (ROS), caused by an imbalance in the body’s antioxidant system, has been related with the pathogenesis of various human diseases, such as cancer and cardiovascular diseases, and inflammation [44]. Antioxidant activity is the most basic biological property of polyphenols, ranging from flavonoids and lignans of small molecules to tannins of higher molecular weight [4,7,45]. Okuda’s early in vitro studies on the antioxidant effects of polyphenols estimated by (1) 1,1-diphenyl-2-picrylhydrazyl (DPPH) radical scavenging test, (2) Cu(II)-catalyzed autoxidation of ascorbic acid, and (3) lipid peroxidation in rat liver mitochondria and microsomes, demonstrated that the antioxidative potencies of ellagitannins, including oligomers, were generally higher than those of small molecular polyphenols, as well as α-tocopherol and ascorbic acid [46]. 

**Figure 7:**
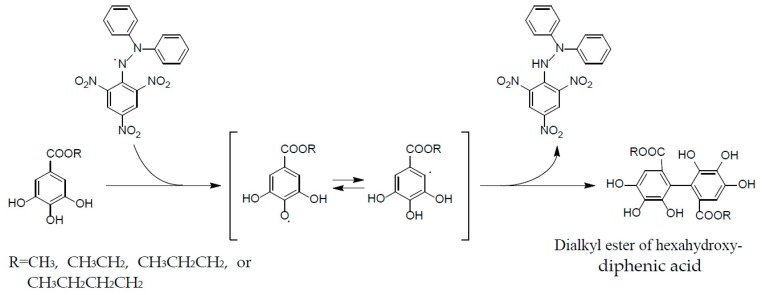
Formation of dialkylester of hexahydroxydiphenic acid in radical reaction of alkyl gallate with DPPH.

These properties are ascribable to the potent radical scavenging ability of ellagitannins, which can terminate free radical chain reaction of other compounds, e.g., lipid peroxidation, by their self-oxidation, thus preventing the oxidation of lipids, proteins, or DNA. A stable radical of an ellagitannin, geraniin (**1**), generated upon air oxidation in an alkaline DMSO solution, was substantiated by the observation of its free radical signals with modulation width 0.05 G in the ESR spectrum [47]. The reactivity of a phenolic radical generated upon donating a phenolic hydrogen radical to another free radical (ROS) was indicated by the treatment of an alkyl gallate with DPPH radical, which produced a dialkyl ester of hexahydroxydiphenic acid by mutual coupling of transient *C*-centered galloyl radicals [48] (Figure 7). This radical coupling reaction is reminiscent of the ellagitannin biosynthesis by laccase-like enzymes, as described earlier. 

The biological antioxidant efficacies of representative condensed tannins, hydrolysable tannins, and related simple phenolics, such as catechin, methyl gallate, and pyrogallol, were also evaluated by cyclic voltammetry. In these studies, the redox potentials of all polyphenols at pH 6‒8 were reported to be substantially below 1000 mV, thus implicating that they act as reducing agents (radical scavengers) for the peroxyl (*E*^−^1000 mV) and hydroxyl (*E*^−^2300 mV) radicals [49].

Although these redox potentials of the tannins were similar to those of simple polyphenols, tannins were 15–30 times more effective at quenching peroxyl radicals than simple phenolics or Trolox (6-hydroxy-2,5,7,8-tetramethyl-chroman-2-carboxylic acid) in a metmyoglobin assay [50]. This result suggests a significance of high molecular weight and the proximity of many aromatic rings and hydroxyl groups for the free radical scavenging ability. 

Antioxidant and anti-inflammatory effects of oenothein B-rich *Epilobium* and *Oenothera* species against oxygen stress have been studied, in order to justify their traditional usages as herbal supplements or tea [11,37,38,51]. *Epilobium* species (willowherbs) have long been used to improve urogenital functions (prostate, bladder, and hormone troubles) in European folk medicine. The extracts of the three most popular *Epilobium* species (*E. angustifolium*, *E. hirsutum*, and *E. parviflorum*), which contained oenothein B (**4**) in high quantities (20–35%), exhibited inhibitory effects on lipoxygenase and hyaluronidase, with IC_50_ around 25 μg/mL and 5 μg/mL, respectively. Additionally, the radical scavenging properties of these extracts were demonstrated by the significant reduction of ROS generated from *N*-formyl-methionyl-leucyl-phenylalanine (f-MLP) and phorbol myristate acetate (PMA)-induced neutrophils, with IC_50_ 5 μg/mL and 25 μg/mL, respectively. A plausible active constituent responsible for these activities was considered as the predominant oenothein B. In fact, oenothein B (**4**) inhibited myeloperoxidase (MPO) release from stimulated neutrophils with IC_50_ 7.7 μM, similarly to the anti-inflammatory drug indomethacin (IC_50_ 15.4 μM), and hyaluronidase with IC_50_ 1.1 μM [39,51]. Similarly, Kiss et al. reported that extracts of *Oenothera paradoxa* and *O. biennis* exhibited anti-inflammatory activity by inhibiting hyaluronidase and lipoxygenase in a concentration-dependent manner, and an inhibitory effect against ROS production from human neutrophils [37,38]. The antioxidant property of extracts from the most common *Epilobium* species, all of which were shown to be rich in oenothein B (**4**) as estimated by LC/MS, were measured by a simple spectrophotometric method using 2,2′-azinobis-(3-ethylbenzthiazoline-6-sulfonic acid) (ABTS), and the extract of *E. parviflorum* was shown to have the highest radical-scavenger activity among these extracts, and comparable to that of well-known antioxidants, Trolox and ascorbic acid [11].

### 5.2. Antitumor Effect

#### 5.2.1. Cytotoxicity against Tumor Cell Lines

Tumor cell growth is regulated in the balance between proliferation and apoptosis. There is considerable amount of evidence indicating that ellagitannins reduce the growth of cancer cells by inhibiting cell proliferation and inducing apoptotic cell death. 

In in vitro studies of macrocyclic ellagitannins performed with cancer cell lines, oenothein B (**4**), woodfordin C (**5**), and cuphiins D_1_ (**8**) and D_2_ (**7**) significantly inhibited the growth of the human oral epidermoid (KB), cervical (HeLa), prostate carcinoma (DU-145), and hepatocellular (Hep-3B) carcinoma cell lines, and the promyelocytic leukemia (HL-60) cell lines, and showed less cytotoxicity than adriamycin against a normal cell line (WISH) [52]. The mechanism for the cyctotoxicity of cuphiins D_1_ (**8**) was examined using HeLa cell lines, and was suggested to be due to induction of apoptosis by inhibition of Bcl-2 expression [53]. Moreover, oenothein B, woodfordin C (**5**) and D (**14**) showed higher cytotoxic activity against human oral squamous cell carcinoma and salivary gland tumor cell lines than against normal human gingival fibroblasts. These cytotoxicities were also indicative of induction of apoptotic cell death as characterized by DNA fragmentation and cleavage of cytokeratin 18 by activated caspase(s) [27,54]. Woodfordin I (**17**) suppressed the proliferation and induced apoptosis in human chronic myelogenous leukemia K 562 cells, which was mediated through the intrinsic mitochondria-dependent pathway [24].

#### 5.2.2. Antitumor Effect Caused by Tumor-Related Enzyme Inhibition

Activity-guided fractionation of the bioactive components of *Epilobium capense* led to the isolation and identification of oenotheins A (**13**) and B (**4**) as potent inhibitors of 5α-reductase and aromatase, enzymes involved in the etiology of benign prostatic hyperplasia. Potencies of the inhibitory effects against 5α-reductase were IC_50_ 1.24 μM for oenothein A (**13**) and IC_50_ 0.44 μM for oenothein B (**4**), respectively, although they were substantially weaker than the positive control finasteride, with IC_50_ 5 nM. On the other hand, against aromatase, oenotheins A (**13**) and B (**4**) displayed 70% and 33% inhibition at 50 μM, respectively, higher and comparable with the synthetic reference compound aminoglutethimide (37% inhibition at 50 μM) [10]. Similarly, oenothein B (**4**) from *E. angustifolium* was found to be specifically able to induce neutral endopeptidase in prostate cancer PC-3 cells, which inactivates growth stimulatory neuropeptides [55]. This enzyme is also known to be involved in prostate cancer progression. Thus, these results might offer a pharmacological explanation for the use (improvement of prostate diseases) of *Epilobium* extract as a folk medicine. As the bioavailability of oenothein B (**4**) still remains unsolved, Kiss et al. further investigated the consistency between the in vitro and in vivo effects of *E. angustifolium* (EA) aqueous extract, using LNCaP human prostate carcinoma cells (in vitro) and rats intraperitonially implanted with LNCaP cells (in vivo) [56]. EA extract (20, 50, 70 μg/mL) and oenothein B (**4**) (2, 5, 10 μM) showed a significant reduction of proliferation of LNCaP cells in dose-dependent manner without affecting the normal human skin fibroblast cells, which was correlated with the induction of apoptosis. These effects were indicated to be comparable to reference compound camptothecin. Similar reduction of the prostatic adenoma up to 13% was observed upon oral administration of EA extract (50–200 mg/kg) to rats implanted with LNCaP cells, suggesting significant and consistent effects in the in vitro and in vivo assays. In order to characterize active metabolites of EA extract (oenothein B) produced by intestinal bacteria, the urinary metabolites obtained from rat and human volunteers supplemented with EA extract were investigated by an application of UHPLC-DAD-MS/MS analysis of ellagitannin metabolites, such as urolithins [57]. Although any bioactive metabolites of oenothein B remained uncharacterized, oenothein B metabolism was suggested to be obviously different from those of other ellagaitannins [56].

In a screen conducted to find inhibitors of DNA topoisomerase-II (topo-II), woodfordin C (**5**) isolated from the leaves of *Woodfordia fruticosa* [14] was shown to inhibit topo-II dose dependently [58]. This in vitro potency was much stronger than those of the clinically used drugs, adriamycin (ADR) and etoposide (ETP). This compound inhibited DNA synthesis, rather than RNA and protein synthesis, in a similar way to ETP. Upon evaluation against cultured human tumor cell lines, woodfordin C (**5**) showed remarkable antitumor activity (IC_50_ 0.07 μg/mL) against PC-1 (lung carcinoma) cells, and moderate activity against MKN 45 (stomach cancer) (IC_50_ 1.73 μg/mL) and KB cells (IC_50_ 5.58 μg/mL), in comparison with ADR and ETP (IC_50_ 0.12–0.67 μg/mL). Also, in vivo antitumor activity of woodfordin C (**5**) against colon 38 (mouse colon adenocarcinoma), subcutaneously inoculated to the flank of a BDF_1_ mouse was shown by 55% inhibition of tumor growth, 16 days after i.v. administration of **5** with 1.5 mg/kg/day (once daily for 5 consecutive days). This result suggested that antitumor mechanism may be through the inhibition of topo-II [58]. It is noted in the literature that **5** may not be taken directly into tumor cells, because of its large molecular mass and a high anionic charge, thus, further studies on bioavailability of **5**, as well as **4**, are still needed. 

Epstein–Barr virus (EBV) is a human B lymphotropic herpes virus known to be closely associated with nasopharyngeal carcinoma (NPC). Inhibitory activity against EBV-DNA polymerase, which is a key enzyme during EBV replication, was estimated for the macrocyclic dimers, eugeniflorin D_2_ (**22**) and oenothein B (**4**), isolated from *Eugenia uniflora*, and the former (**22**) exhibited a remarkable inhibition with IC_50_ 3.5 μM, while the latter (**4**) showed weaker activity with IC_50_ 62.3 μM in comparison with a positive control, phosphonoacetic acid (EBV replication inhibitor) (IC_50_ 16.4 μM) [40]. 

On the other hand, degradation of poly(ADP-ribose) on specific chromosomal proteins in eukaryotic cells, mainly by poly(ADP-ribose) glycohydrolase, is considered to be an important factor in the regulation of gene activation, DNA replication and transcription, and cell death [59]. In a search for potent and specific inhibitors of poly(ADP-ribose) glycohydrolase purified from human placenta, oligomeric ellagitannins were found to be more potent inhibitors (IC_50_ 0.3‒7.1 μM) than condensed tannins and monomeric hydrolysable tannins (IC_50_ 15.5‒31.8 μM), and also than the previously known inhibitors, daunomycin and ethadridine (IC_50_ 50‒100 μM), and cAMP (IC_50_ 5‒10 mM) [60]. The most potent inhibitory activity was exhibited by nobotanin K (a tetramer from *Tibouchina semidecandra*; Melastomataceae [61]) and oenothein B (**4**), with IC_50_ 0.3 and 1.8 μM, respectively, on a molar concentration basis. It is notable that the condensed tannins (epicatechin gallate dimer to tetramer) and flavan-3-ols tested were not active even at 100 mM. As depoly(ADP-ribosyl)ation of chromosomal proteins was suggested to be involved in the initiation of glucocorticoid-sensitive mouse mammary tumor virus (MMTV) transcription, the inhibitory effect of oenothein B (**4**) on MMTV gene expression in intact 34I cells, derived from C3H mouse mammary carcinoma, was examined. As a result, pretreatment with oenothein B (**4**) potently suppressed, dose-dependently in a range of 1‒50 μM, the induction of MMTV mRNA by dexamethasone (100 nM). It is noteworthy that oenothein B showed no inhibitory effect against other poly(ADP-ribose) metabolizing enzymes tested, such as poly(ADP-ribose) polymerase and NAD^+^ glycohydrolase, even at 0.5 mM, thus indicating that oenothein B is specific for poly(ADP-ribose) glycohydrolase [62]. Although poly(ADP-ribosyl)ation has been suggested to be involved in regulation of DNA repair, transcription, centrosome duplication, and chromosome stability, the regulation of the degradation of poly(ADP-ribose) and its significance remain less understood [59,63,64]. Therefore, oenothein B (**4**) may not only be a promising therapeutic candidate, but also one of the useful chemicals in pharmacological experiments for elucidation of the physiological role of poly(ADP-ribose).

#### 5.2.3. Host Mediated Antitumor Activity

Detailed investigations on the host-mediated antitumor effect of ellagitannins and other related polyphenols were reported by Miyamoto and Okuda’s group [65,66,67]. Oenothein B (**4**) exhibited remarkable host-mediated antitumor activity upon intraperitoneal (ip) injection several days before or after inoculation of sarcoma-180 (S-180) tumor cells into the abdomen of mice [65]. The evaluation of the activity was evaluated by the number of survivors (in six mice/group), and the percent increase in life span (%ILS) 60 days after administration. Treatment with a 10 mg/kg dose of oenothein B (**4**) four days before inoculation of S-180 resulted in four survivors out of six mice, and 196 %ILS, demonstrating that oenothein B was the most potent among the approximately 100 polyphenols tested, including condensed tannins and ellagitannins (monomers‒tetramers), as well as related small molecular polyphenols, such as caffeic acid derivatives and gallotannins. Oenothein A (**13**) and woodfordin D (**14**) showed 102.7 and 123.0 %ILS, respectively, and each with one survivor. Oenothein B also exhibited anticancer activity against murine mammary carcinoma MM2 in C3H/He mice by i.p. administration at 10 mg/kg dosage, at one, four, and seven days after the cancer inoculation. The effect evaluated after 60 days showed high %ILS (126.8%), and four survivors out of six mice [63]. This effect was stronger than that of OK-432, a streptococcal preparation with a potent immunostimulatory activity [68]. in vivo treatment with these antitumor-active dimers induced cytotoxic adherent peritoneal exudate cells, including stimulated macrophages producing and secreting interleukin (IL)-1β [65,67,69]. Cuphiin D_1_ (**8**) was also shown to stimulate human peripheral blood mononuclear cells (PBMCs) and release, dose-dependently, IL-1β, IL-2, and TNF-α, and then activate T cells. Therefore, cuphiins D_1_-activated T cells via IL-1β, in vitro, might account for the host-mediated mechanism of 8. Thus, the antitumor effect of these tannins was attributed to the enhancing of the immune response of the host, and not due to their direct cytotoxic action on tumor cells [70].

### 5.3. Immunomodulatory Effect

In order to understand the mechanisms underlying diverse biological activities of macrocyclic ellagitannins, such as anti-inflammatory, antitumor, and antimicrobial effects, the immunomodulatory effects of oenothein B (**4**) have been investigated in various in vitro or in vivo immune systems. 

Oenothein B (**4**) was reported [71] to activate a number of phagocyte functions in an in vitro evaluation using neutrophils and monocytes purified from healthy human blood, resulting in the induction of intracellular Ca^2+^ flux, production of ROS, NF-ĸB activation, and proinflammatory cytokine production. On the other hand, intraperitoneal administration of **4** to female BALB/c mice induced significant levels of keratinocyte, which directly correlated with the neutrophil influx into the peritoneum. However, the oenothein B-related small molecular weight polyphenols, gallic acid, pyrogallol, pyrocatechol, and 3,4-dihydroxybenzoic acid, were all inactive, suggesting the necessity of the whole structure of **4** for the modulation of the phagocyte functions, both in vitro and in vivo [71]. Oenothein B (**4**) was also shown to reduce, dose-dependently, nitric oxide (NO) production, inducible nitric oxide synthase (iNOS) mRNA, and iNOS protein levels, without inhibiting the iNOS enzymatic activity in lipopolysaccharide (LPS)-stimulated murine RAW 264.7 macrophage cells [72,73]. The IC_50_ value of **4** for inhibition of inducible NO production was 17.7 μM, while gallic acid, a component unit of **4**, showed much weaker activity, with IC_50_ 631.6 μM, implying the requirement of the entire structure of **4** for this effect, in agreement with the above results [71]. The inhibition of inducible NO synthesis by **4** in a dose-dependent manner was also observed in Toll-like receptor (TLR)-stimulated RAW 264.7 cells, which were stimulated using TLR4 and TLR2 agonists. Such an inhibitory effect by **4** was shown to be NF ĸB-dependent, but independent from the interferon (IFN)-γ/JAK-STAT pathway [72]. As inappropriate or excessive NO production by iNOS is closely associated with numerous inflammatory diseases and neuropathic pain states, oenothein B (**4**) might be a promising lead for the development of therapeutic agents as the effective inhibitors of NO production. Ramstead et al. reported that oenothein B (**4**) stimulated innate lymphocytes, including bovine and human γδ T cells and NK cells, resulting in either increased CD25 and/or CD69 expression. Oenothein B thus enhanced the production of IFNγ by bovine and human NK cells, and also by human T cells. These responses were not observed with other commonly studied polyphenols. Since IFNγ is known to contribute to antitumor, antibacterial, and antiviral cell responses, these data suggested an additional mechanism for the immune-enhancing properties of oenothein B [74]. Innate immune cell responsiveness is known to be affected by aging. Then, the responsiveness of oenothein B (**4**) in T cells from individuals over a broad range of ages (cord blood, young, and adult donors) was estimated by measuring IFNγ production, and clear differences depending on the ages were observed, that is, oenothein B (**4**) induced IFNγ production in T cells from adult humans and cattle, but not in T cells from human cord blood and bovine calves [75]. 

Recently Yoshimura et al. [76] reported the significant immunomodulatory effects of oenothein B (**4**) on human dendritic cells (DCs), which are widely present in various tissues in contact with the external environment, such as the skin, nose, lungs, stomach, and intestines, and have critical functions in the initial immune response as antigen presenting cells. Oenothein B (**4**) had significant immunoregulatory effects on DCs through suppression of cell surface molecules, downregulation of cytokine production, and induction of their apoptosis. When oenothein B (**4**) (25 μM or 100 μM) was added to the cultured immature DCs (iDCs) supplemented with TNF-α (75 ng/mL) and LPS (100 ng/mL), the expression of cell surface molecules, CD1a and CD83, was shown to be suppressed significantly at 100 μM of **4**, resulting in the dysfunction of DC-mediated immune responses by the inhibition of cell maturation and subsequent antigen presentation. The suppressive effect on DCs was shown to be due to the induction of apoptosis by a flow cytometric assay. However, in the apoptosis induced by **4**, none of caspase-3/7, 8, and 9, which play crucial roles in cell apoptosis, was activated, suggesting a caspase-independent mechanism for this apoptosis. Morphological change of tannin-treated DCs was confirmed by fluorescence microscopy, showing significant nuclear condensation without DNA fragmentation, similar to that of AIF (apoptosis-inducing factor)/PARP [poly (ADP-ribose) polymerase]-dependent cell death [77]. Oenothein B also markedly suppressed the production of inflammatory cytokines, such as IL-1β and IL-6, in a dose-dependent manner at 25 mM and 100 mM. Plant tannins, including condensed tannins, are generally considered to be stable in acidic conditions [78], and thus could travel unmodified, or form complexes with some inner biomacromolecules, such as proteins and dietary fiber, through the pharyngeal tube and stomach, until metabolized in the small intestine. These effects on DCs may thus be significant in the traditional usages of oenothein B- or related ellagitannin-containing medicinal plants for the treatment of a variety of inflammatory diseases.

Apart from inflammatory effects in peripheral tissues, in vivo effects of oenothein B (**4**) on the damage to the central nervous system due to systemic inflammation was reported by Okuyama et al. [79]. Peripherally injected LPS is reported to induce a depressive-like abnormal behavior through induction of microglial immune responses in the brain of mice [80]. In an open-field test using mice treated with LPS (i.p.) (1 mg/kg mouse), orally administered (p.o.) oenothein B (300 mg/kg) showed significant increase of the locomotive activity at 24 h after LPS treatment, compared with that of the control group with depressive-like behavior. Immunohistochemical and biochemical investigation of oenothein B-administered mice indicated suppression of LPS-induced microglial activation and LPS-induced cyclooxygenase-2 production in the hippocampus and striatum of these mice. These results suggested that oenothein B has the ability to reduce neuroinflammation in the brain during systemic inflammation. Since oenothein B itself might hardly pass through the blood–brain barrier (BBB), some metabolites of 4 produced by intestinal microflora [57,81,82] were considered to likely affect the peripheral inflammation, which was followed by the suppression of the inflammatory responses in the brain, although the possibility that these metabolites can pass through BBB and act directly in the brain as anti-inflammation agents was not excluded. 

### 5.4. Antimicrobial Effects

Antimicrobial effects of ellagitannins, including antibacterial, antivirus, and antiprotozoal activities, have been documented in many papers and reviews, including those by Okuda, Haslam, and Kolodziej [4,6,46,83,84]. Among them, a notable activity was the synergistic effects of certain polyphenols with currently used antibiotics against drug-resistant bacteria. Many pathogenic bacteria, such as methicillin-resistant *Staphylococcus aureus* (MRSA), have acquired resistance to various clinical antibiotics. This worldwide problem is likely driving the development of new antibiotic drugs in an endless stream. Synergistic effects of ellagitannins, including oenothein B (**4**) and tellimagrandin I (**2**), with β-lactam antibiotics (e.g., oxacillin), were found to restore the effectiveness of these antibiotics against MRSA. When used together with these tannins, the MICs of oxacillin against MRSA strains were markedly lowered to 1/250 or 1/500 [85]. These results may provide one strategy for overcoming emergent bacterial resistance.

## 6. Conclusions

Since the discovery in 1990 of oenothein B (**4**) and woodfordin C (**5**), a unique class of dimeric ellagitannins with macrocyclic structures, many analogous ellagitannins (oenotheins, woodfordins, cuphiins, eugeniflorins, and oenotherins), including oxidized oligomers up to heptamer with molecular weight 5488, have been isolated from various medicinal plants belonging to Onagraceae, Lythraceae, and Myrtaceae. Their novel structures were elucidated by spectroscopic analyses (ESIMS, 1D and 2D NMR, CD) and chemical degradation. Oenothein B is commonly the most abundant constituent in plants containing this class of macrocyclic ellagitannins. 

Oenothein B (**4**) and its analogs were documented to possess diverse in vitro and in vivo pharmacological properties, including antioxidants, antitumor, immunomodulatory, and antimicrobial effects, and their potencies were, in general, much higher than those of the related polyphenols with small molecular weight, suggesting the necessity of the entire structure of tannins for exhibiting activities. Hence, this type of oligomer may provide promising leads for the development of novel therapeutics and chemopreventive agents. An often-claimed problem is that high molecular weight tannins (polyphenols) have a limited bioavailability in biological systems due to their low solubility, stability, and membrane permeability. Therefore, biological activities of tannins and related polyphenols found in in vitro and in vivo assays have to be interpreted with caution, as noted in many papers or reviews, for the necessity of further studies. Increased interest for the fate of ellagitannins in the gastrointestinal tract has thus prompted investigations on the bioavailability or actual metabolites of ellagitannins in detail [55,56,57,81,82,86], and these aspects were reviewed by Tomas-Barberan et al. [87] and Torronen [88]. On the other hand, an oral delivery device, which encapsulates oenothein B or other ellagitannins, was reported for their enhanced protection through the gastrointestinal tract [89]. Further studies on these matters, including different manners of intestinal metabolism from those of non-macrocyclic ellagitannins, are strongly encouraged, for a better understanding and effective usage of these bioactive macrocyclic ellagitannins.

## Figures and Tables

**Table 1:** Distribution of oenothein B and related macrocyclic oligomers in plants.

Family Species	Tannins	Ref.
Oenotheraceae		
*Oenothera erythrosepala* Bordas	oenothein B	[9]
*O. biennis* L.	oenotheins A, B	[20,37,38]
*O. laciniata* Hill.	oenotheins A, B, D, F, G	[12]
*O. tetraptera* Cav.	oenotheins A, B, oenotherins T_1_, T_2_	[25,26]
*O. paradoxa* Hudziok	oenothein B	[38]
*Epilobium capense* Buch.	oenotheins A, B	[10,11]
*E. angustifolium* L.	oenotheins A, B, woodfordin I, tetramer–heptamer	[10,11,22,24,39]
*E. pyrricholophum* Franch. et Sav.	oenotheins B	[39]
*E. hirsutum* L.	oenotheins B	[10,39]
*E. palustre* L.	oenotheins A, B	[39]
*E. dodonoei* Vill.	oenothein B (HPLC) *	[10]
*E. stereophyllum* Fres.	oenothein B (HPLC)	[10]
*E. salignum* Hausskn.	oenothein B (HPLC)	[10]
*E. parviflorum* Schreb.	oenothein B (HPLC)	[10]
*E. roseum* Schreb.	oenothein B (HPLC), (LC/MS) *	[10,11]
*E. tetragonum* L.	oenothein B (LC/MS)	[11]
*E. montanum* L.	oenothein B (HPLC), (LC/MS)	[10,11]
Lythraceae		
*Lythrum anceps* Makino	oenothein B	[31]
*Woodfordia fruticosa* Kurz.	oenotheins A, B, woodfordins C, D, E, F, I	[13,14,20,21]
*Cuphea hyssopifolia* Humb.	oenotheins A, B, woodfordin C, cuphiins D_1_, D_2_	[16]
*Punica granatum* L.	oenothein B	[34]
Myrtaceae		
*Eugenia uniflora* L.	oenothein B, eugeniflorins D_1_, D_2_	[15,40]
*Melaleuca leucadendron* L.	oenothein B	[5]
*Myrtus communis* L.	oenothein B, eugeniflorin D_2_	[28]
*Eucalyptus alba* Reinw. Ex Blume	oenothein B	[33]
*E. robusta* Sm.	oenothein B, eugeniflorin D_2_, eurobustin C	[27]
*E. cypellocarpa* LAS Johnson	oenothein B, eugeniflorin D_2_	[27]
*E. globulus* Labill.	oenothein B	[30]
*E. consideniana* Maiden	oenothein B	[32]
*E. viminalis* Labill.	oenothein B	[32]
*E. pulverulenta* Sims.	oenothein B (HPLC) *	[30]
*E. nicholii* Box Hill. Merbourne	oenothein B (HPLC)	[30]
*E. camaldulensis* Dehnh.	oenothein B (HPLC)	[30]
*Myrtus communis* var. *microphylla* Willk.	oenothein B (HPLC)	[30]
*Austromyrtus dulcis* L.S. Sm.	oenothein B (HPLC)	[30]

* Method for identification, characterization, or detection.
